# Inadequate Social Housing and Health: The case of Oliver Bond House in inner-city Dublin

**DOI:** 10.12688/openreseurope.16767.1

**Published:** 2023-11-28

**Authors:** Lidia Katia C. Manzo, Hannah Grove

**Affiliations:** 1 Languages, Literatures, Cultures and Mediations, University of Milan, Milan, Lombardy, Italy; 2 Department of Geography and Maynooth University Social Sciences Institute, Maynooth University, Maynooth, Ireland; 3 School of History and Geography, Dublin City University, Dublin, Leinster, Ireland

**Keywords:** Social Housing, Health, Care, Inadequate Housing, Mental Health, Public Health, City of Care approach, Health in All Policies, Mould, Dublin, Financialization of Social Housing, Urban Restructuring, Urban Poor Communities, Women Residents’ Activism

## Abstract

**Background:**

Inadequate housing is an important social justice issue that adversely affects health.

**Methods:**

Drawing on an extended ethnography case study, this paper presents the results of a resident-led survey to highlight the health consequences of inadequate social housing, as residents wait for a ‘fair regeneration’ of their social housing ‘flats’ estate within a gentrifying inner-city Dublin neighbourhood.

**Results:**

Four key concerns were identified by residents as part of this analysis: (1) substandard housing conditions which are physically harmful to health; (2) the emotional toll of an unsafe social environment; (3) lack of child friendly and community green spaces; and (4) constrained mobility due to inaccessible housing design.

**Conclusions:**

The results highlight the urgent need to place greater priority on the maintenance of the existing social housing stock and demonstrate the need for public housing policies that recognize the quality and quantity of adequate housing provision, where care is at the heart of housing policies. The paper also presents a novel ‘City of Care’ framework, following the need to develop an ethics of care within cities where public health, community wellbeing, solidarity, residents’ empowerment, and social justice principles are at the forefront. Given that housing is an essential contributor to good health, it is now time for a joint public housing and public health agenda to create healthier homes by confronting the everyday impact of inadequate housing to tackle social inequalities more broadly

## Plain language summary

Emerging research from the EU-funded CITY-OF-CARE project illuminates the health impacts of inadequate housing, particularly public and social housing, where the most vulnerable people live.

Four key concerns were identified by the residents as part of this analysis: (1) substandard housing conditions that are physically harmful to health, (2) the emotional toll of an unsafe social environment, (3) lack of child-friendly and community green spaces, and (4) constrained mobility due to inaccessible housing design.

Results demonstrate the need for public housing policy which ensures long-term strategic engagement between housing and health improvement sectors and the need to adopt a ‘City of Care’ approach, which emphasizes the significance of social capital, affordable/adequate housing and community infrastructure. Placing ethics of care at the heart of all policies will ensure that public health and social justice are at the forefront.

## Introduction

An adequate standard of living is a major determinant of (public) health and well-being ( Bonnefoy, 2007; Krieger & Higgins, 2002) and a human right protected under international law ( UN, 1948). While adequacy is determined in part by social, economic, cultural, climatic, ecological, and other factors, the UN (1991) recognizes that adequate housing means having adequate privacy, adequate space, adequate safety (from toxic hazards), adequate security (from crime/anti-social behaviour), adequate physical habitability, lighting and ventilation, adequate basic infrastructure, adequate location with regard to access to services, and the opportunity to be physically active, all at a reasonable cost. Thus, the concept of “adequacy” is particularly significant, since it serves to underline several factors (physical or material, social and psychological) which must be taken into account in determining whether particular forms of housing can be considered “inadequate.”

Furthermore, housing is experienced at various scales (e.g., the individual, family, neighbourhood, community, and political levels) and according to the dimensions of existing socio-economic inequalities, as shown by Borrell *et al*. (2023). This is particularly so when it comes to public and community housing (collectively known as “social housing”) where welfare retrenchment, product deregulation and financial liberalization each contributed to a dual process of residualised social housing targeting only those tenants in the greatest need (disability, poor physical health, mental illness, old-age, lone mothers, exiting prison or risk of homelessness).

**Figure 1.**
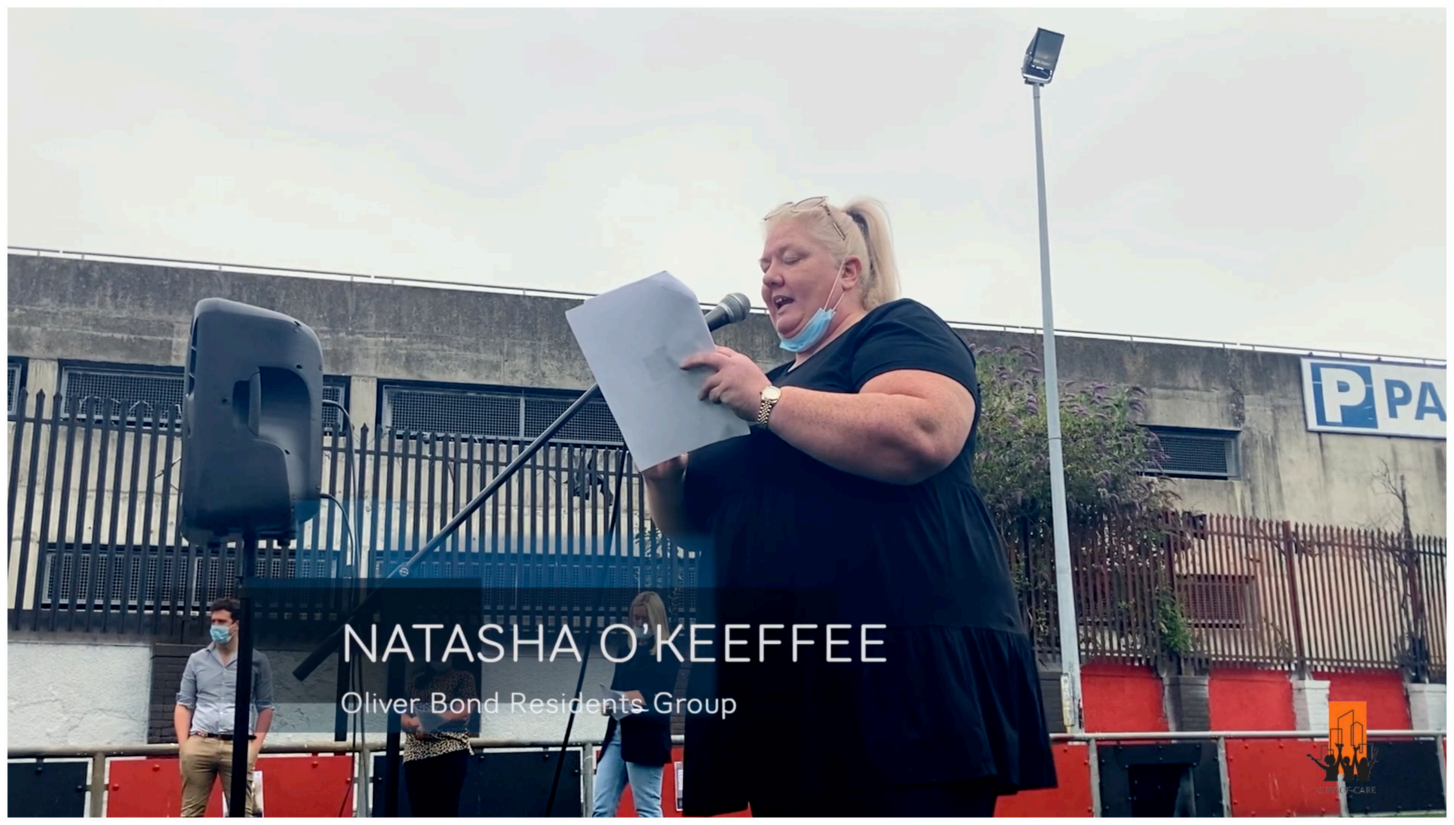
Oliver Bond residents demand accelerated regeneration: Natasha O’Keeffee delivering her 'We're sick of waiting!' media campaign speech on June 30, 2021. Video credits to Lidia K. C. Manzo for CITY-OF-CARE. Written informed consent obtained from image subject.

This paper presents the outcomes of the EU-funded CITY-OF-CARE project ^1^, which draws on extended participatory ethnographic research to analyse a social housing community’s journey in seeking the fair regeneration of their homes in inner-city Dublin. A strategy of resistance ( De Certeau, 1984) was developed through collaboration with community development partners, residents, and academics, which included a ‘Sick of Waiting’ media campaign (see Figure 1) ^2^, a survey, and a collaborative workshop to fill the data gap of residents’ experiences of the health impact of living in inadequate housing. This paper presents an analysis of this 2022 survey. Results demonstrate the need for public housing policy which ensures long-term strategic engagement between housing and health improvement sectors and the need to adopt a ‘City of Care’ approach, which emphasizes the significance of social capital, affordable/adequate housing and community infrastructure ( Manzo, *forthcoming b*).

## How does inadequate housing affect our health?

Inadequate housing is an important social justice issue that adversely affects health. Its impact on health is multifaceted, encompassing both direct and indirect tangible and intangible dimensions ( World Health Organization, 2018; p.3). Housing quality can have a tangible influence on health through factors such as dampness, cold temperatures, mould, and heat; however, it also exerts a softer, more social influence by shaping feelings of belonging, attachment, and ontological security ( Shaw, 2004).

Living in inadequate housing fundamentally disrupts the functions of social contexts, leading to a reduced sense of "ontological security" ( Giddens, 1992) and biographical continuity that typically underpins confidence in life's stability. Ontological insecurity refers to a feeling of vulnerability and instability that arises when individuals have no control over their living conditions. This sense of insecurity can be intensified by factors such as the type of housing tenure and stress stemming from an inability to manage or fix housing issues ( Byrne & McArdle, 2022).

Understanding the consequences of inadequate housing requires the consideration of broader social and environmental determinants of health. These consequences ripple across various levels, affecting individuals and households and extending beyond the confines of the home to impact neighbourhoods and communities. The risks associated with substandard housing vary. They encompass physical injuries resulting from hazardous conditions as well as respiratory and cardiovascular health deterioration due to mould and poor air quality ( Bonnefoy, 2007). Overcrowding in inadequate housing also increases the risk of exposure to infectious diseases ( WHO, 2018) and is associated with poor mental health outcomes. Stress and anxiety can be exacerbated by living in poor housing conditions, leading to increased social isolation, either because of fear or challenges related to leaving the home, or the avoidance of visitors. Households living in social housing are at risk of poorer health outcomes because of structural issues related to overcrowding and poor quality housing, including damp walls, leaks, poor ventilation, and inadequate light and heating systems ( Russell *et al*., 2021; Suglia, 2018). This is particularly distressing and challenging when the responsibility for repair lies with others who may not fulfil be obligated ( Byrne & McArdle, 2022).

Recognizing that the deterioration of housing conditions, often through 'managed decline,' inflicts gradual and long-term harm to people's health and overall well-being, akin to what has been termed 'slow violence' ( Pain, 2019), it becomes apparent that addressing the housing crisis extends beyond a mere focus on infrastructure. This framework should not only account for how housing affects health but also shed light on the extent to which the interaction between health conditions (including health histories, diagnoses, and prognoses) on one hand, and the institutions and economic markets within the housing system on the other, can be broadly described as “health selective” ( Smith, 2012, p. 42). This selectivity can manifest in different ways, such as in favour of individuals with health-related housing needs rehousing them into the social rented sector, or more critically, by favouring health conditions for access to the majority of tenure, such as mortgage-backed owner-occupation.

The connection between housing and care highlights the profound impact of housing conditions, markets, and governance on shaping care opportunities. Care embodies our collective capacity to create the necessary political, social, material, and emotional conditions for the well-being and prosperity of most individuals and living beings on the planet ( Gilligan, 1982; Tronto, 1993). In the following section, we explore how care should form the foundation of all policies with a particular focus on health policies. We advocate a participatory approach to policymaking that prioritizes the voices and needs of individuals residing in inadequate housing and their respective communities.

## Why should we care about inadequate social housing and who has a responsibility to care?

In this study, we leverage Smith's (2005) work to argue that care, as both an ethical responsibility and a foundational value system, should be central to all policies. Building upon Tronto's (1993) concept of ‘giving care,’ this entails a profound responsibility for performing caregiving tasks and ensuring their fulfilment, especially in the realms of policy and care provision. Daly (2021) emphasized the significance of conceiving care as a practice, acknowledging its operation within a broader political context, as observed by Gilligan (1982). This context raises questions concerning the allocation of public resources, including housing, as well as the considerations of fairness, justice, and responsibility. It is crucial to acknowledge that ethical considerations of care are inherently context specific. More recently, Lynch (2022) stressed the importance of care, framing it not only as an ethical matter, but also as a question of justice within and beyond capitalist systems. Both care- and rights-based perspectives on justice are considered essential, with the COVID-19 pandemic highlighting their complementary nature.

Care operates through various practices and scales within these domains, as is evident in Smith's ( 2005, 2012) and Smith *et al*.’s (2003) research. Within this framework, Power (2019) highlighted the intricate link between housing and resident care, with housing conditions, markets, and governance playing key roles. In this context, the 'City of Care' approach highlights the strong connection between need and vulnerability within social housing communities. Individuals facing various challenges, including physical, emotional, or psychological vulnerabilities, often find these issues intersecting with sociocultural and economic disadvantages. To tackle such complex challenges effectively, it is crucial to establish a participatory framework in social housing policy that addresses hierarchical relationships and power imbalances. This approach should prioritize respect for residents' dignity, voice, and rights within the city, encompassing both housing and health rights, while also promoting responsibility and responsiveness toward both the urban environment and its inhabitants ( Till, 2012).

## Dublin’s social housing context

Dublin is currently grappling with a housing crisis, marked by a scarcity of high-quality, affordable housing to accommodate the city's burgeoning population. According to a 2019 report from the Central Statistics Office, the population of Dublin is projected to increase by as much as 31.9% by 2036 ( Central Statistics Office, 2019). 

Existing studies have raised concerns regarding the age, quality, suitability, and habitability of the Irish social housing stock ( Grotti *et al*., 2018; Russell *et al*., 2021). The European Committee of Social Rights (ESRC) have found the Irish state to be in violation of human rights and their responsibilities, by failing “to take sufficient and timely measures to ensure the right to housing of an adequate standard” for those individuals living in Local Authority housing ( ESRC, 2017). Furthermore, individuals living in poor-quality housing or in unsafe conditions face elevated health and safety risks during lockdowns worldwide ( UN, 2020).

Issues of housing and inequality have been deeply intertwined, and pre-existing systemic inequalities have already been evident prior to the pandemic, impacting all facets of society ( Borrell *et al*., 2023). Governmental funding cuts, dating back to the 1980s, have intensified the pressure to redevelop or privatize social housing throughout Europe ( MacLaran & Kelly, 2014). This has further worsened the health and social consequences of reduced investment in housing provision and maintenance ( Hearne, 2020) in oppressed working-class communities in Dublin ( Bissett, 2023). Consequently, neoliberal policies have reshaped housing, framing it as an “individual responsibility, investment, and asset” ( Power & Mee, 2020; p. 496), failing to prioritize community well-being.

Housing and inequality are mutually reinforcing, as noted by Aalbers (2022) and James *et al*. (2022). Health disparities among different socioeconomic groups are not inherent but rather result from policy failings, making health inequalities are “anything but natural” ( Smith, 2012, p. 43). These inequalities are exacerbated by the careless interplay between politics, markets, and institutions (ibid.), a phenomenon linked to global neoliberal restructuring ( Aalbers, 2016). The key aspect of this neoliberal approach is the absence of an ethics of care, which fails to recognize care as a universal need given that individuals are inherently fragile, vulnerable, and at various stages of life, both givers and recipients of care ( Tronto, 1993). Care has been systematically stripped from housing and urban policies, leaving those most vulnerable to being directed towards and stuck in inadequate and unsuitable housing conditions ( Smith, 2012).

### Oliver Bond House in inner-city Dublin

**Figure 2.**
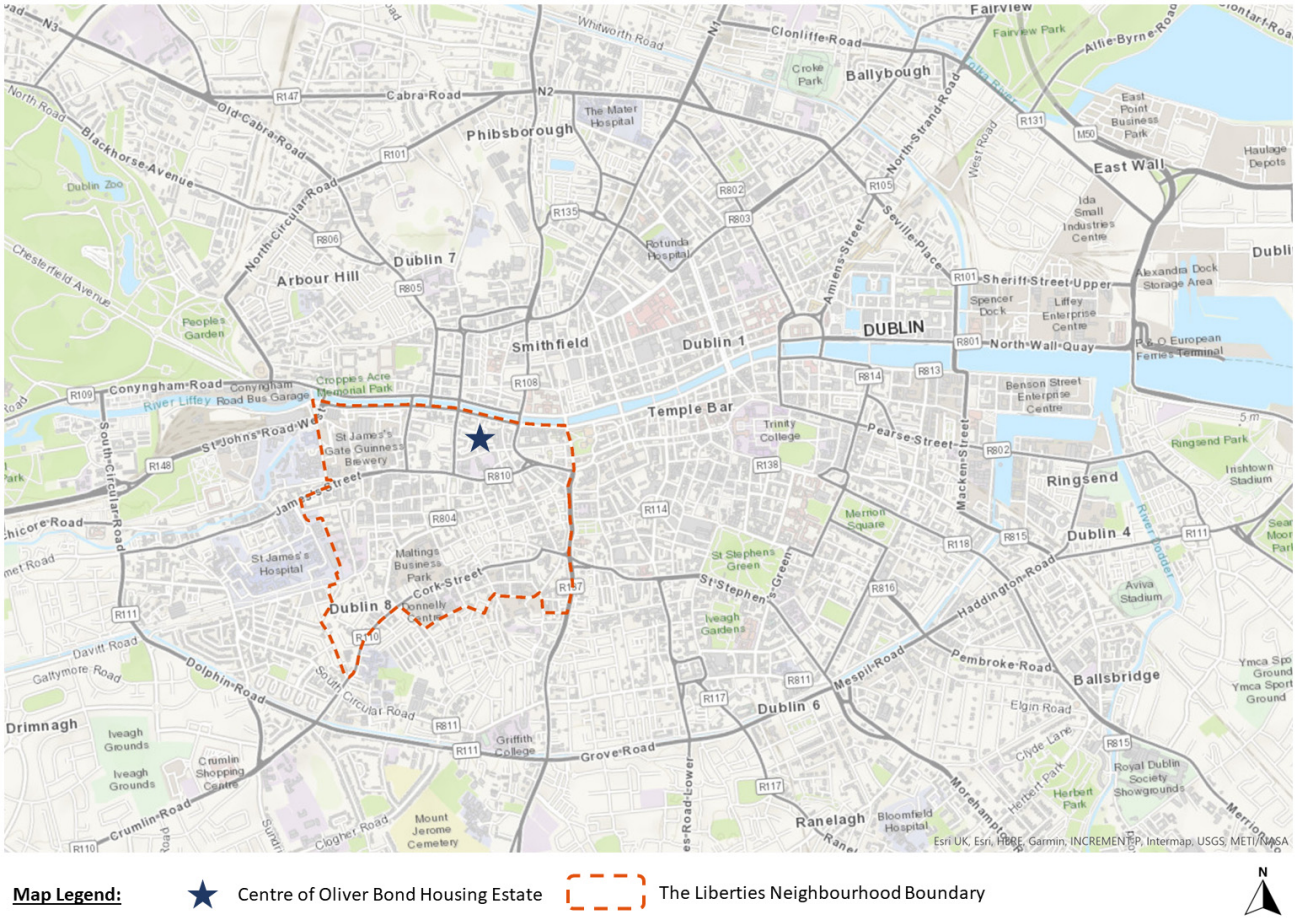
Map of Dublin showing the location of Oliver Bond housing and location of The Liberties neighbourhood (Source: Map produced by Grove (2023) using ArcGIS Online).

**Figure 3.**
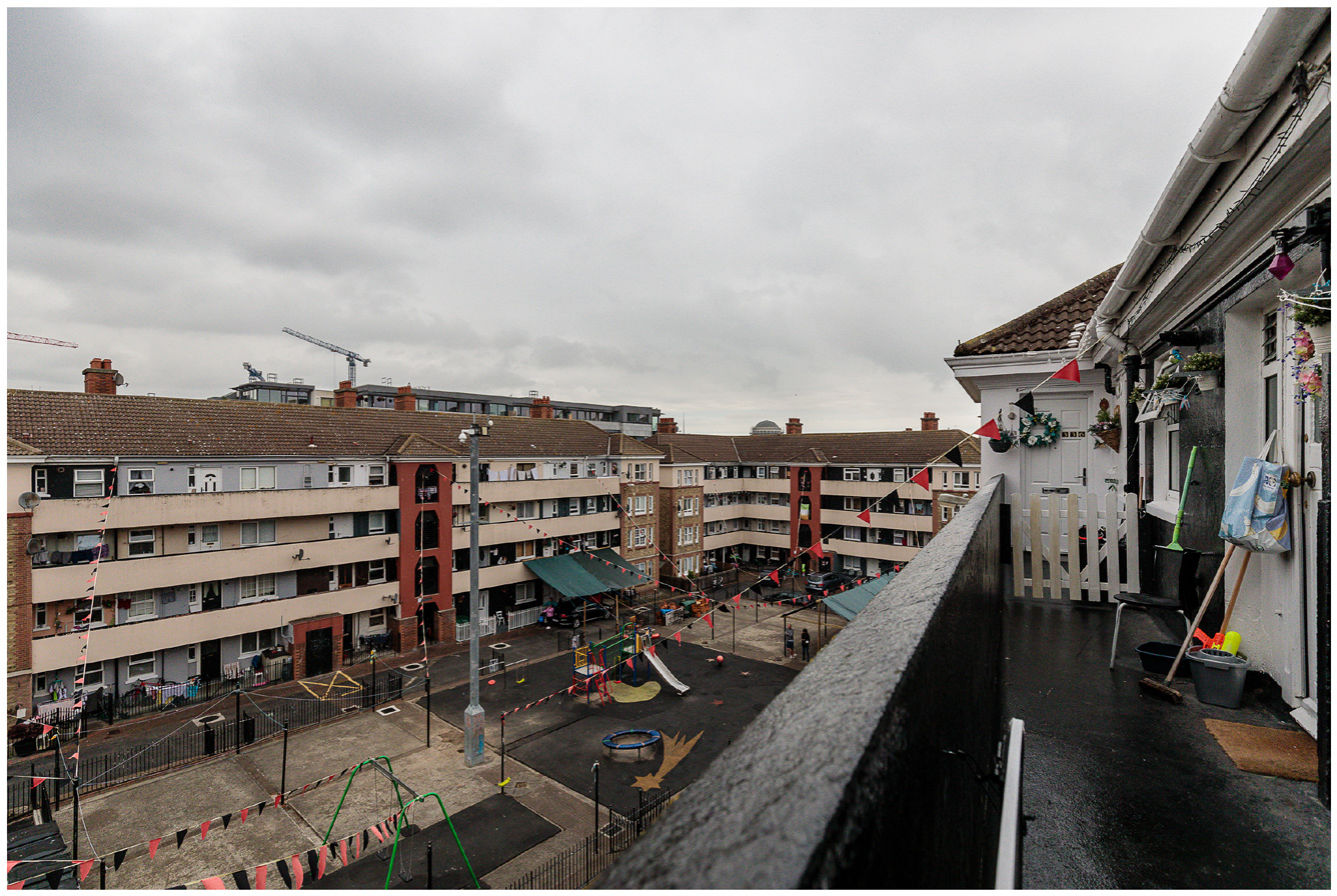
Oliver Bond House in the Summer of 2021: an internal play area built entirely without green spaces. Photo credits to Pierluigi Cattani Faggion for CITY-OF-CARE.

Framed by a city-of-care approach, this research draws on extended ethnography to document the journey of the Oliver Bond community in advocating for fair regeneration of their social housing estate (see Figure 2 and Figure 3) in Dublin's gentrifying inner-city neighbourhood, The Liberties. 

The housing blocks designed and constructed by Dublin Corporation ^3^ during the twentieth century are remarkable achievements of Irish architectural modernism ( Roley, 2011).

Constructed in 1936 on a site formerly occupied by a brewery in Dublin’s Southwest Inner City, the Oliver Bond complex is one of Ireland's most extensive local authority housing estates, comprising 16 distinct blocks of flats and three houses, accommodating a total of 391 households. Spanning an area of 2.847 hectares, this estate currently houses approximately 1,200 residents. More than 10,000 people lived through these flats. Rearing families and working in the local industries and businesses, they have played a significant part of the fabric of daily life in Dublin’s Inner City in the past and today. Many families who arrived in 1936 still live there today. The establishment of this large city-centre flat complex was a response to the 1932 Irish Housing Act, which aimed to address pressing issues related to slum clearance and public health in the city at the time. The buildings themselves were a testament to their architect, Herbert Simms, who designed them along with 17,000 other social housing units across Dublin between 1923 and 1931. Simms' flat blocks, beyond their architectural significance, played a pivotal role in maintaining the urban population at the city core. This strategy retained the existing communities and contributed to the ongoing vitality of city life. The approach involved relocating residents from slums to these new dwellings while also constructing additional blocks as the remaining slum buildings were cleared. Simms himself favoured central block housing as a solution for large-scale slum clearance, in contrast to suburban schemes ( McManus, 2018).

However, the Oliver Bond Complex has faced a pressing need for refurbishment and improvement over the years. Household size, particularly in the context of Oliver Bond's housing units, underscores a significant issue. An average two-bedroom flat within the complex measures only 48 square meters, falling considerably short of the 70 square meters standard for a new two-bedroom flat. Consequently, a majority of families in the complex are considered 'underhoused.' The age of these flats, coupled with associated problems, such as dampness and unreliable services, introduces challenges to the day-to-day functionality of households. These issues, in turn, adversely affect the physical and mental health of residents, as well as their overall dignity ( Watt, 2021). Additionally, residents face chronic underservice when it comes to suitable recreational areas for children and young adults as well as adequate parking, waste disposal facilities, and shared spaces. Moreover, the social profiling of the complex is readily evident in online search results for 'Oliver Bond House,' which differs from similar online searches for other housing developments in Dublin. An ongoing cycle of anti-social behaviour, stigmatization, lowering of morale, and sense of despondency are other key contributory factors in community media ‘defamation’ ( Manzo, 2022a). 

Alongside the level of neglect and decline of Oliver Bond flats, ethnographic research has found evidence of an enduring social fabric and strong community spirit. The material and cultural support that absorbs, sustains, and socializes members of Dublin's Oliver Bond social housing community is provided by networks of kin, friends, and more or less formal local institutions and associations, particularly from the Robert Emmet community development project. These ‘networks of care’ ( Manzo, 2022b) grab people together, giving them dignity.

## Methods

This study is part of a larger research project, the EU Horizon 2020 Marie Skłodowska-Curie CITY-OF-CARE study, which aims to investigate the extent to which women’s networks of care enhance social capital in urban social housing communities facing severe economic and social deprivation due to the financialization of social housing in European cities with diverse socio-political contexts and welfare regimes ( Manzo, *forthcoming a*). In the context of this project, the first author conducted extensive ethnographic and participatory research in the Oliver Bond social housing community within Dublin's Liberties.

To support this methodological approach, the project adopted a "personal network" perspective ( Manzo, 2021) to understand the dynamics and significance of interconnected care providers within close-knit vulnerable urban communities. Combining personal network analysis and visualization with ethnographic and participatory techniques allows for the creative integration of qualitative inquiry and network analysis. This approach is rooted in the idea that researchers build relationships with the community over extended periods, enabling the characterization of social ties. It directly measures an individual's capacity to access various resources within their community network and whether they know someone with access to those resources, representing the multiple dimensions of social capital. Personal networks are vital for the daily functioning of households and play a critical role in managing crises and coping with various stresses, particularly in vulnerable social housing communities ( Chua *et al*., 2011). Notably, the majority of participants were women, reflecting the gendered nature of family care, the prevalence of gender division of labour, and its significant role in low-income communities ( Bettio & Sansonetti, 2015). Women in such communities tend to have extensive family ties and actively connect with their neighbours and extend kin ( Fischer, 1982). Consequently, their networks are centred around tightly knit contexts and are guided by norms of diffuse reciprocity, trust, and commitment, a concept referred to as "networks of care" in this research ( Manzo, 2022b).

Furthermore, the CITY-OF-CARE research project aligns with Burawoy's (2005) call for public sociology that bridges professional and policy sociology with critical sociology. The aim is to address the growing gap between the sociological ethos and the world being studied. In other words, this study encourages academics to engage with issues of significant public and political concern, including debates on public health policy, housing activism, community development goals, and the influence of data and technology on creating healthier homes and cities. By actively participating in field research, the first author dedicated her efforts to supporting the Oliver Bond community in the quest for improved living conditions. She assisted members of their Regeneration Forum in designing the "Health and Community" Survey and analysed its results.

In June 2021, supported by the Robert Emmet community development project, women residents launched the ' we e are Sick of Waiting' campaign ( Manzo, 2022c), urging the Dublin City Council (DCC) to implement immediate housing improvements and fast-track the formation of the Oliver Bond Regeneration Forum ^4^. In 2022, forum members conducted a resident-led survey that addressed topics such as demographics, health, housing's impact on well-being, COVID-19 effects (which will be discussed in another paper ( Grove & Manzo, *forthcoming*), community safety, crime, police interaction, and maintenance. In this article, we examine preliminary evidence from a resident-led survey that established a connection between housing conditions and health. Specifically, we focused on whether residents felt that where they lived impacted their health.

A total of 192 respondents completed the survey ^5^ across 391 households in Oliver Bond, resulting in a 49% response rate. Those who completed the surveys were mostly women living alone or with single parents, reflecting the flat’s demographic composition. Participants had lower educational attainment, with the highest levels of education at either the primary or the secondary level. They also typically worked in manual unskilled labour, were unable to work for health or disability reasons, or were retired. In the survey, 65% of the participants described their health as excellent, very good, or good. Fifteen% reported bad health, and 20% reported that their health was fair. Alongside this, 41% of the respondents identified that a member of their household had a long-term illness or disability. Several participants identified chronic respiratory health conditions, with six reporting that they had Chronic Obstructive Pulmonary Disease (COPD), two had emphysema, eight had asthma, and three had general breathing difficulties.

Open-ended survey questions were coded and categorized into key concerns using content analysis ( Saldaña *et al*., 2011; Weber, 1990) in NVivo 14, Computer-Assisted Qualitative Data Analysis Software (CAQDAS). Four key concerns were identified by the residents as part of this preliminary analysis: (1) substandard housing conditions that are physically harmful to health, (2) the emotional toll of an unsafe social environment, (3) lack of child-friendly and community green spaces, and (4) constrained mobility due to inaccessible housing design.

## The health impacts of inadequate social housing in Oliver Bond

### Key Finding: 65% of residents reported that inadequate housing conditions were negatively impacting their health and wellbeing

**Figure 4.**
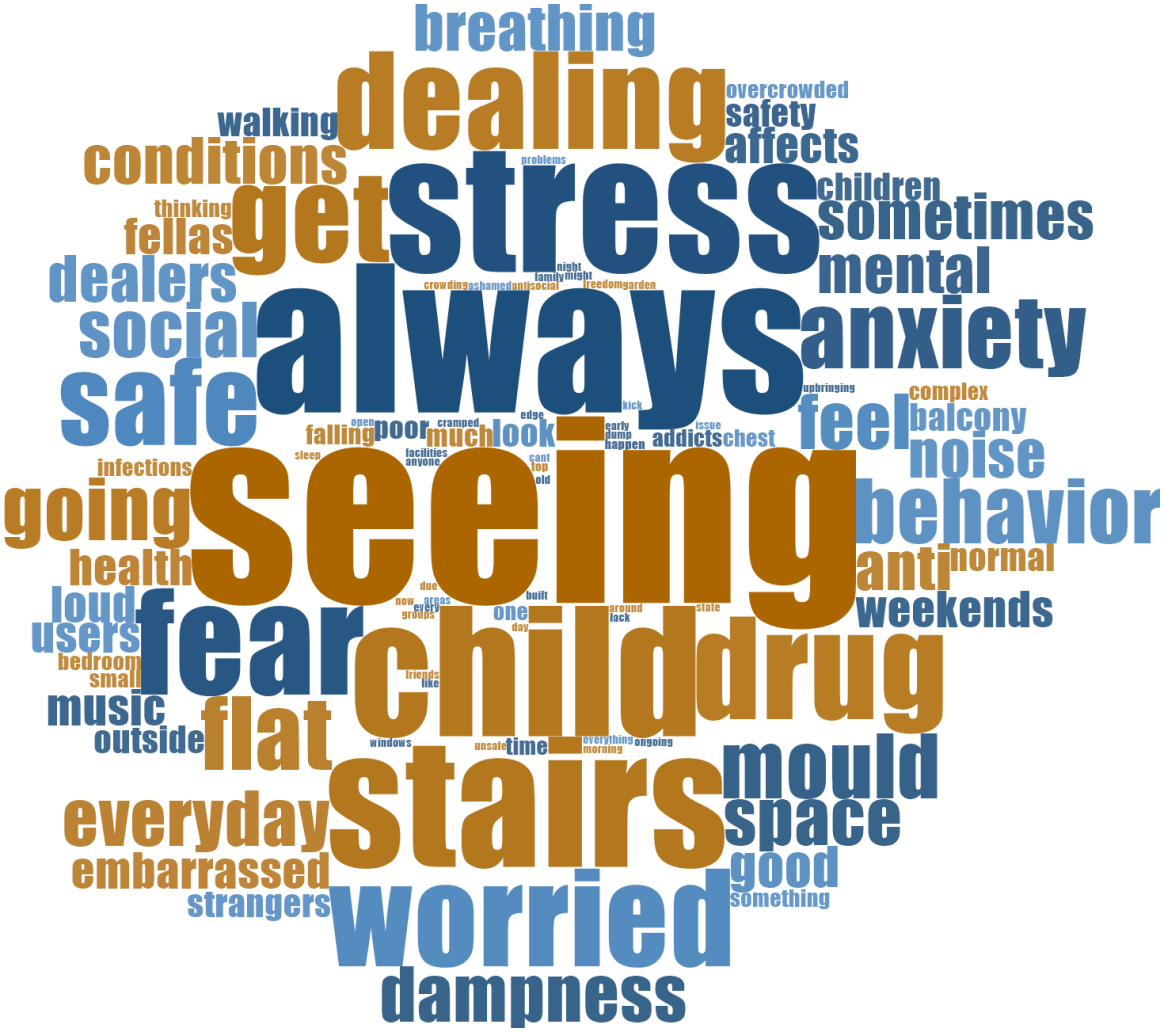
How inadequate social housing is impacting resident health and wellbeing. Source: Manzo and Grove (2023).

Participants were asked whether their living conditions impacted the health of their families, and almost two-thirds (65%) of the residents answered yes to this question. Figure 4 shows a word cloud of the open-ended written responses provided by 100 residents, summarizing how inadequate housing impacted their health.

This word cloud provides a sense of the language used by the residents in the written responses, revealing a strong belief that inadequate housing significantly affects health and well-being. Phrases like "stress," "anxiety," and "fear" underscore the emotional toll, while "safety" and "affects" signify concerns about security. "Health" and "dampness" link housing to physical health problems. "Child" and "behaviour" suggest children are especially affected. Words such as "always," "everyday," and "sometimes" convey the daily struggle, including issues like "noise" and "mould." The mention of "dealers" hints at potential community-related problems stemming from inadequate housing.

**Figure 5.**
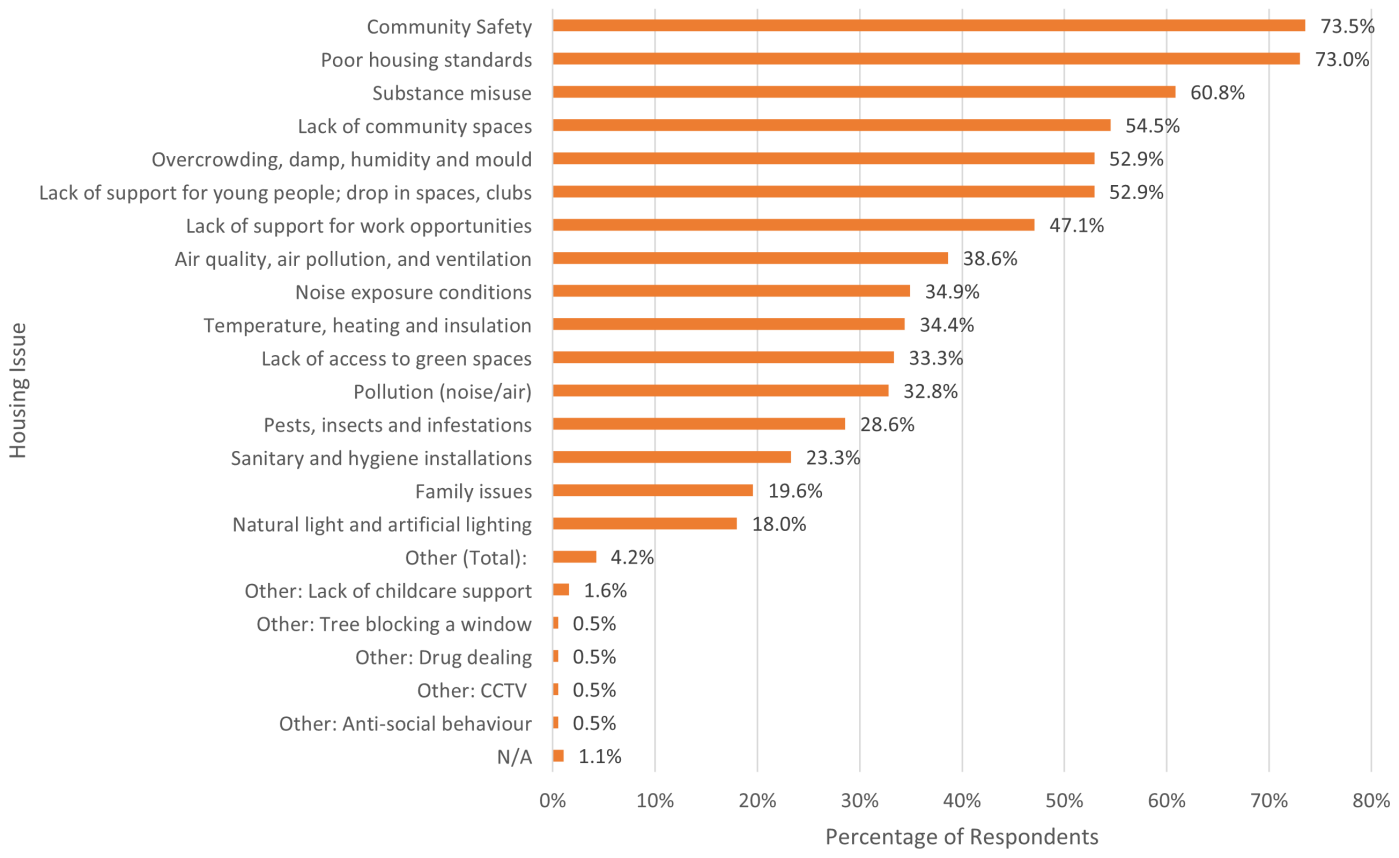
Percentage of residents who identified each housing issue to be impacting their wellbeing. Source: Manzo and Grove (2023).

Alongside the written component, when asked to select housing issues that impacted their well-being (in a select all that applies question). The most commonly selected by residents were community safety (74% identified this as an issue), poor housing standards (73%), concerns about the impact of substance misuse (61%), lack of community spaces (55%), and overcrowding, damp, humidity and mould issues (53%). Figure 5 below provides full list of issues and the percentage of residents who selected them.

### KEY CONCERN 1: Substandard housing conditions which are physically harmful to health

***73% reported poor housing standards and 52.9% reported overcrowding, damp, humidity or mould issues in their home***

The prevalence of mould, damp, poor ventilation, leaks, and cold conditions within the home were identified by residents within the open-ended written responses. Several respondents attributed these conditions to difficulties in breathing as well as contributing to recurrent chest infections.

*“[My] children [have] constant infections and chest problems”*
*“Mould affects my breathing”*
*“Poor ventilation and mould not good for breathing”*
*“Mould and damp get chest infections”*

**Figures 6 and 7.**
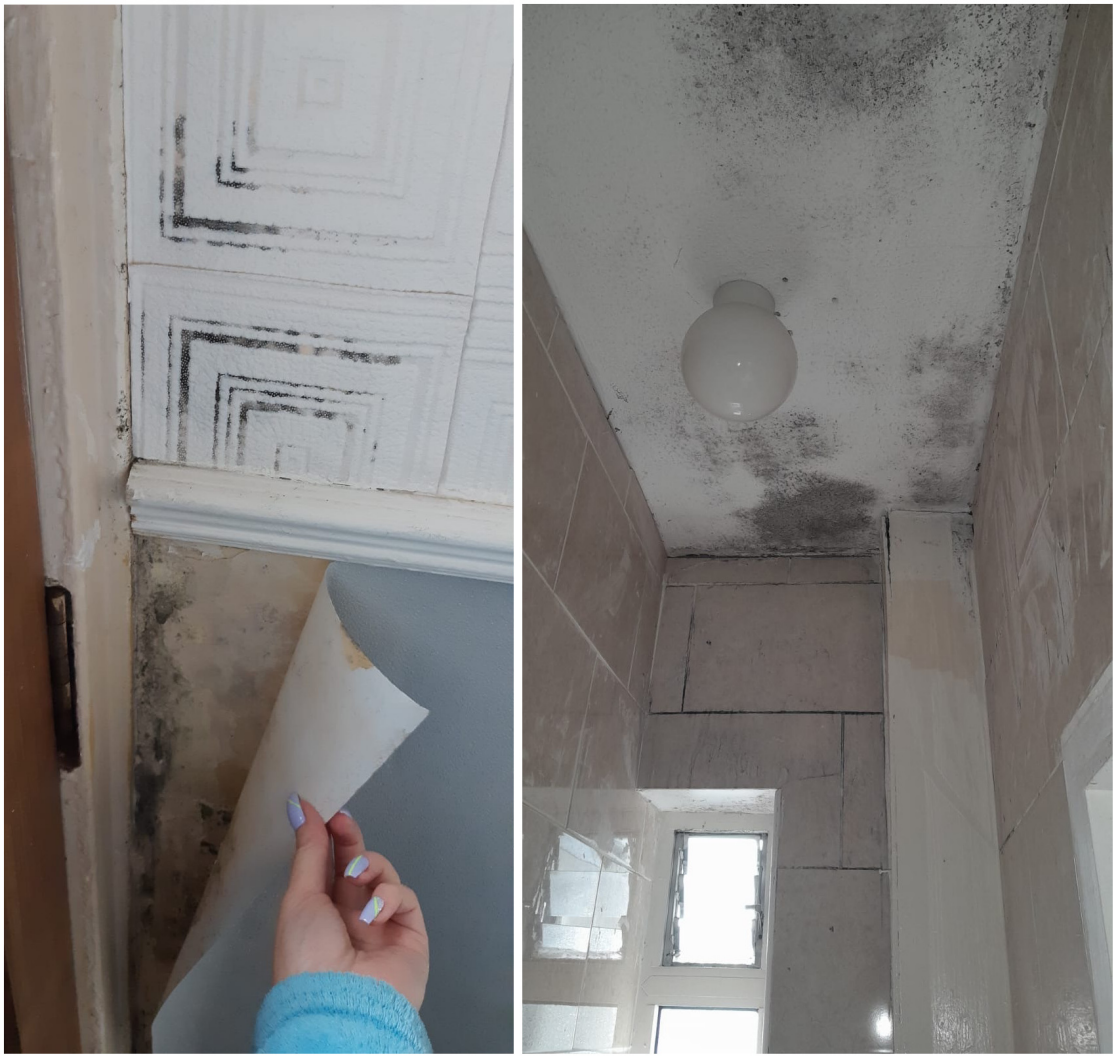
Mould in Oliver Bond House flats in the summer of 2022. Source: Photographs provided by residents.

Figures 6 and 7 present photographs taken by Oliver Bond House residents, which provide insight into some of the mould issues experienced in their housing.

Overcrowded and cramped conditions and a general lack of space were identified as problems within housing:

*“Living in cramped conditions, dampness and overcrowded”*
*“My 13 yr old son having to share a room with his 8yr old sister”*
*“Overcrowding, unhealthy conditions - no space for time out”*
*“...stressful in closed space with us all here, too small”*

One participant reported that they were currently sleeping on a sofa. In addition, this participant commented on the presence of pests, such as ‘silver fish’ and slugs. Although there was only one open-ended response that identified pests, the presence of pests was selected as an issue by 29% of respondents.

Existing research has demonstrated the negative impacts of poor indoor air quality, inadequate ventilation, and overcrowding on respiratory health outcomes (see Wimalasena *et al*., 2021), particularly among children. Disproportionately, it is children living in rented (both public and private) housing that are most at risk ( Holden *et al*., 2023).

### KEY CONCERN 2: The emotional toll of an unsafe social environment

***73.5% of residents had concerns about community safety and 60.8% were concerned about substance misuse outside their homes***

Another key concern among participants was related to safety concerns. This was primarily due to concerns about drug use in general and drug dealing within communal areas, with many participants describing a constant stream of people coming in and out of the flat complex to buy drugs:

*“Drug users [are] always in and out - [it is] stressful. Sometimes [I] dread weekends - noise and loud music from fellas”*
*“Looking out at dealing every day not good [for] mental health”*
*“Look out [of my] balcony, [I am] just seeing dealing 24/7 - strangers in and out”*
*“Both myself and my husband suffering with stress and anxiety due to the lack of safety”*

Several participants were fearful about the crime and anti-social practices their children were observing and experiencing as well as the normalization of this:

*“Constantly worried about what my child [is] seeing, thinking it's normal”*
*“Kids have to see drugs all the time”*
*“My kids see too much [of] what happening in the flats, they [are] selling drugs in front of the kids”*

Residents raised general concerns about anti-social behaviour, crime, and violence. Noise concerns were also raised, particularly the presence of parties and loud music, resulting in residents being unable to sleep.

Participants commonly reported the emotional and mental health tolls brought about by these challenges. In particular, they identified the stress, fear, worry, depression, and anxiety caused and exacerbated by inadequate housing conditions and persistent safety concerns. What was particularly striking about the participant accounts was the language used to describe the relentlessness of these challenges. The words “constant” and “always” were repeatedly mentioned when describing concerns. Many participants reported that they were on guard, on the edge, or waiting for something bad to happen, highlighting that many residents are in a constant state of stress or anxiety and are unable to relax in their homes.

*“I am always on guard - something might kick off”*
*“Constant flow of strangers passing through - [you are just] waiting [for] something to happen”*
*“… safety concerns, on edge when kids out”*
*“Constant fear worsens my anxiety and depression”*

Existing studies have shown that perceived stress, particularly in chronic and enduring forms, can affect poor health, both directly and indirectly ( Kaplan *et al*., 2013).

Respondents spoke about the social impacts of inadequate housing, with some residents feeling too ashamed to invite friends and family members to their houses.

*“Can’t bring anyone around. [I am] embarrassed and ashamed”*
*“Don't get to see family or friends as much as I'm embarrassed and don't feel safe where I live”*

One participant reported that their child experienced social anxiety. Two residents raised concerns about walking past drug dealers or walking alone.

*“Fear. Walking by ourselves at night or early morning”*
*“I can get anxious walking through flats - [I am] worried by dealers”*

*These findings suggest that many residents curtail their social interactions with others, owing to shame and embarrassment, and also avoid leaving the home at certain times due to fear of crime. The overall impact of this is that inadequate housing leads to reduced opportunities for social interactions within the immediate local environment, which has also been observed by Palmer *et al.* (2005).*

There were additional questions specifically related to crime and community safety; however, these were not included, as they will be discussed in a subsequent paper ( Manzo & Grove, *forthcoming*).

### KEY CONCERN 3: Lack of child-friendly and community green spaces

***54.4% reported a lack of community spaces with 33.3% referring specifically to a lack of green spaces***

**Figure 8.**
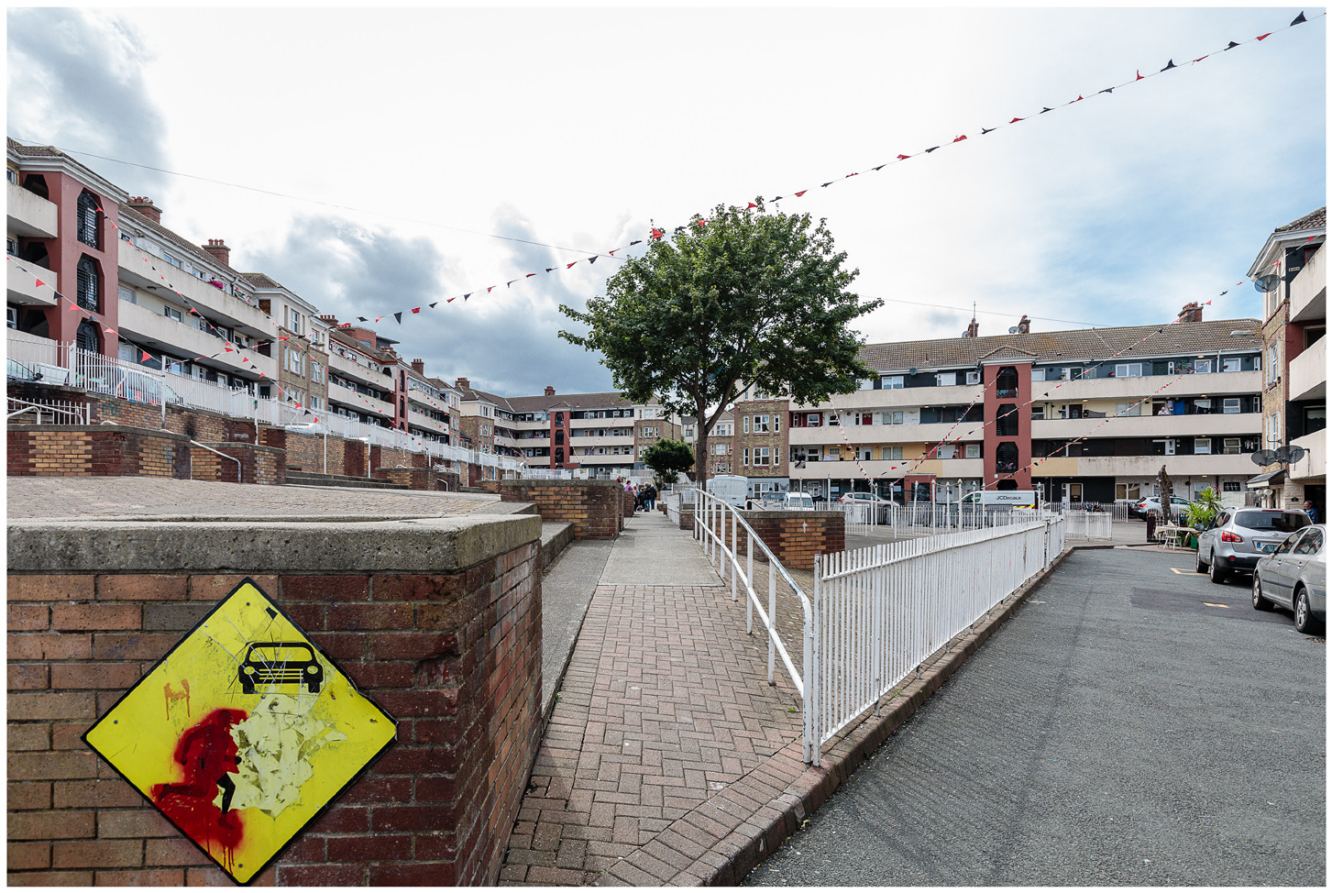
The courtyard of Oliver Bond House in the Summer of 2021: a detailed view of its concrete design, with limited green spaces. Photo credits to Pierluigi Cattani Faggion for CITY-OF-CARE.

Participants spoke about the inadequate provision of green spaces, a general lack of greenery within the social housing complex and open areas, the lack of activities and facilities for children, and the lack of freedom arising from not having a garden (see Figure 8). Another participant spoke about the built-up nature of the complex, coinciding with their fear of going outside.

*“There are no green spaces for my children”*
*“Looking at concrete”*
*“I don't have any freedom like a garden”*
*“No facilities for the young - [it’s] tough on them”*
*“Built up, no open areas, fear of [going] outside”*

**Figure 9.**
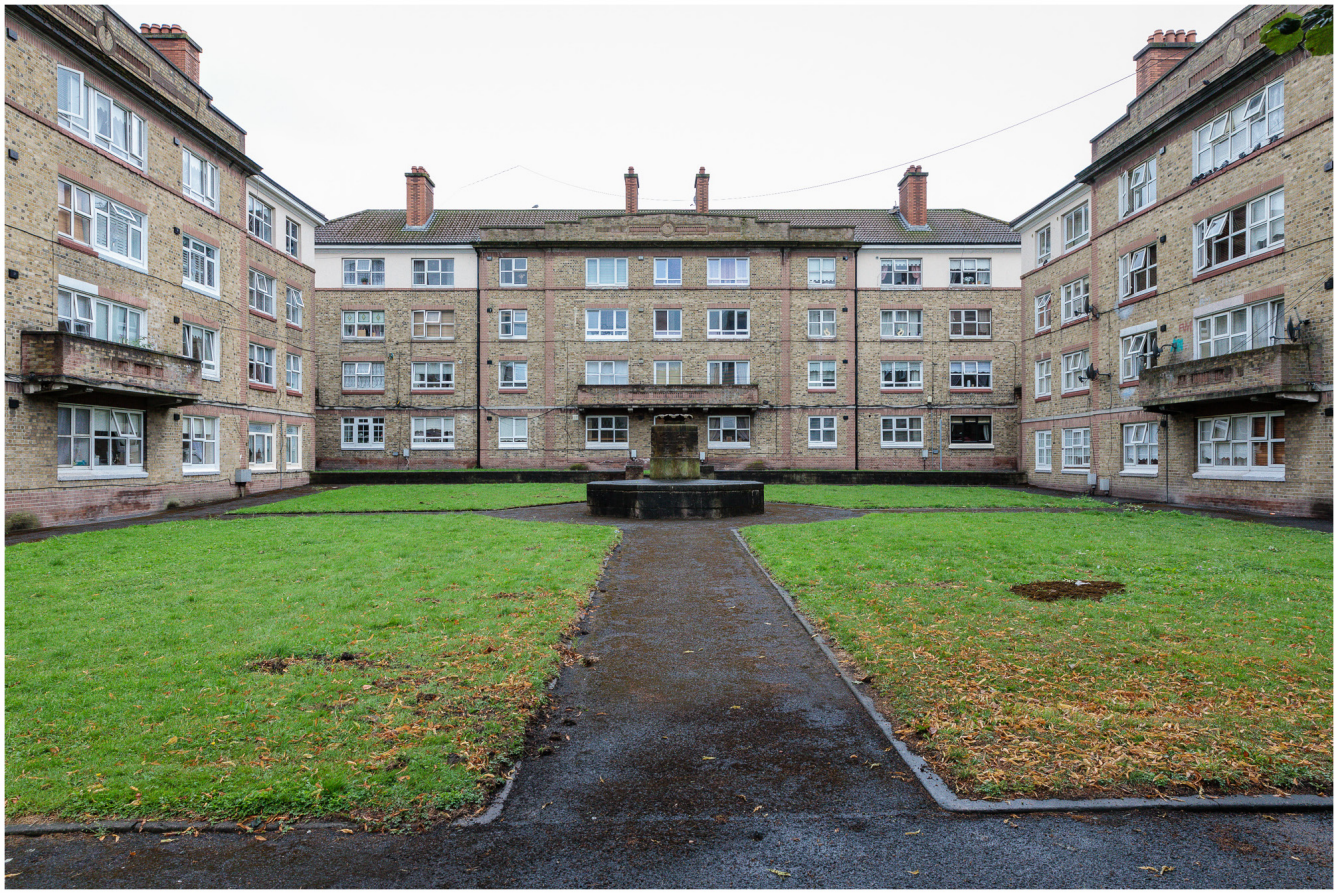
Oliver Bond House in the Summer of 2021: exterior detail on Usher Street. Photo credits to Pierluigi Cattani Faggion for CITY-OF-CARE.

Simm's designs revolutionized housing block access and circulation, as seen in the Oliver Bond House. Access was through courtyards via decks with wide and shallow flat layouts. Public activities shifted to the open courtyard, acting as a surveillance mechanism, and were enclosed by plain concrete facades, diverging from the decorative street front. However, these green spaces remain underused ( Figure 9).

There was an emphasis on the impact of this lack of community infrastructure on children, in particular, as well as the safety concerns described in the previous section.

As a result, residents expressed concerns about letting their children play outside and raised concerns about their upbringing in general. This was related to both the combined impacts associated with the inadequate provision of child-friendly spaces and the safety concerns arising from anti-social behaviour:

*“[It’s] not safe [to] let [my] child out”*
*“I worry about my child growing up”*

One participant stated that their children did not feel safe as a result of the anti-social behaviour in communal spaces, while another reported that their child had social anxiety.

**Figure 10.**
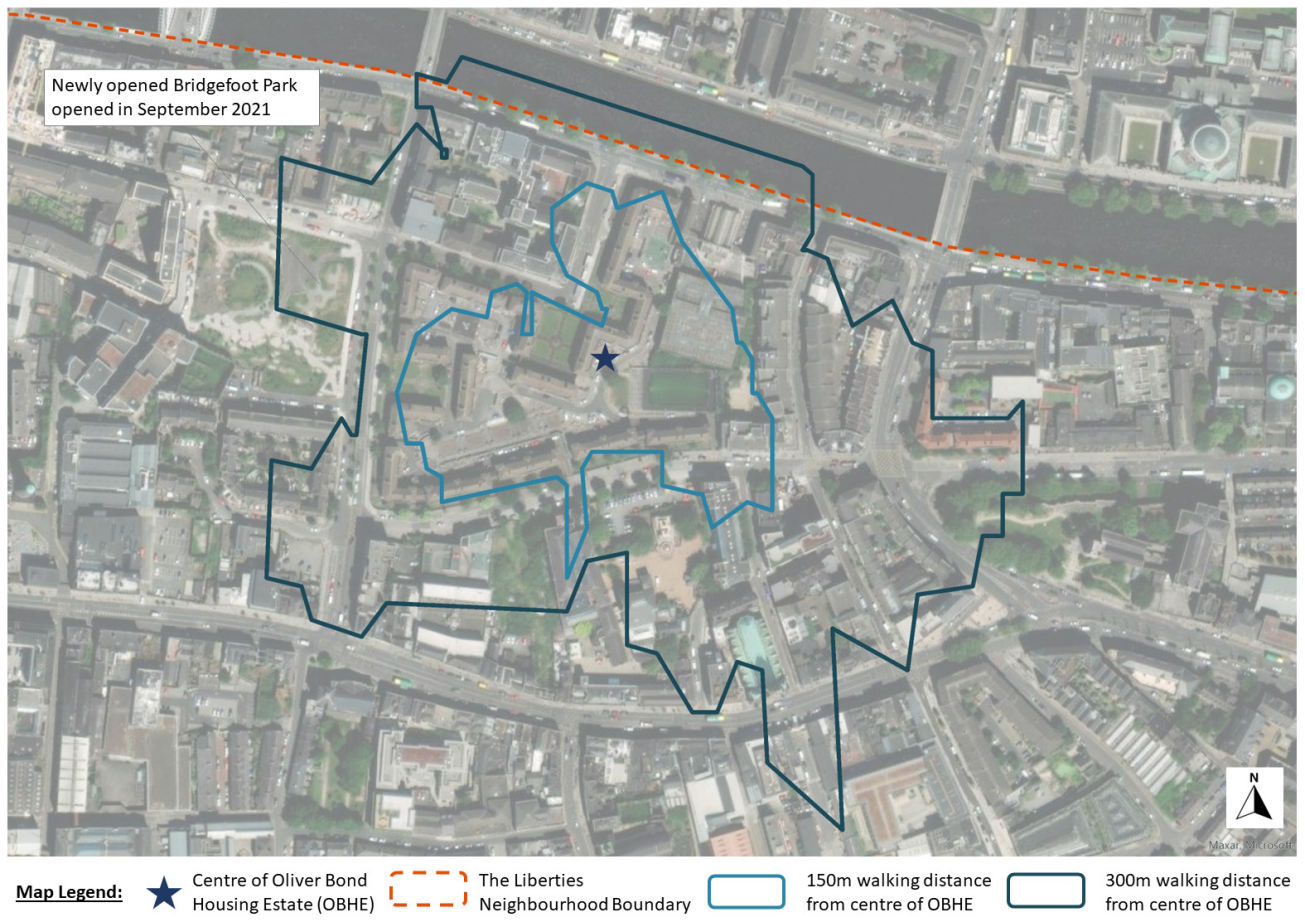
Map showing what is available at approximately 150m and 300m distances from the centre of Oliver Bond Housing Estate (Source: Map produced by Grove (2023) using ArcGIS Online).

Dublin 8, where the Oliver Bond is located, has been identified as an area deficient in green spaces and trees (see Clavin *et al.*, 2021). Recent research projects have co-produced a community-led greening strategy, recognising the importance of this strategy for health and well-being ( Mapping Green Dublin, 2021). Building on this strategy and since the resident survey has been completed, a new park at Bridgefoot Street was designed and opened in May 2022. This park is approximately 300 m from the centre of the Oliver Bond Housing Estate (see Figure 10). While this has gone some way to address the green space deficit in the area, the housing estate still lacks more immediate places for children to play safely.

### KEY CONCERN 4: Constrained mobility due to inaccessible housing design

**Figure 11.**
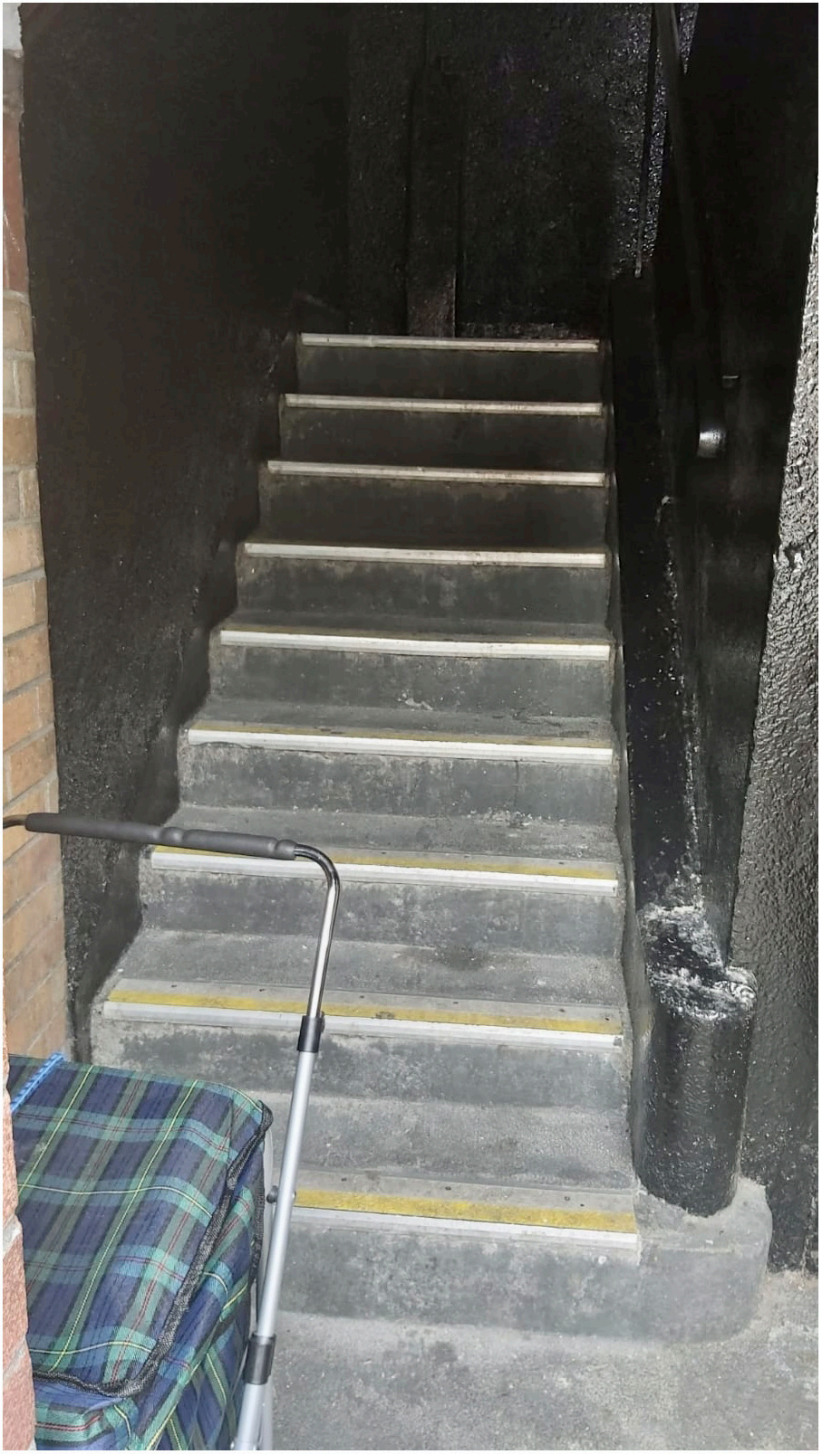
Photograph of staircase in Oliver Bond Social Housing Complex. Source: Photographs kindly given to the authors by the residents.

A key problem identified with the social housing complex was the stairs, which was the most common code across the written data. Similar to many cost-effective social housing units designed by Herbert Simm in 1930s Ireland, Oliver Bond House features a distinctive four-storey flat-roofed block. It is characterized by its dual sides: a more ornate public-facing elevation and a cement-rendered private side adorned with continuous access galleries. However, in contrast to the 1930s perimeter blocks that harmonized with the existing urban layout, the current presence of open staircases along the balconies without any protection (see Figure 11) makes them challenging to access on one hand by older people or people with mobility challenges, and susceptible to intrusion by individuals with anti-social behaviour. A number of participants found the stairs particularly challenging to navigate, and some residents were unable to leave their homes as a result. This was particularly the case for older residents who lived on the top floor of the 3-storey complex and those with health and mobility challenges.

*“Getting older now, stairs an issue”*
*“On top of the block, can't go downstairs”*
*“Partner, weak heart going up and down stairs, no lift”*

Figure 11 shows the staircases that exist within this social housing complex.

The provision of barrier-free (step-free) housing is an important component of housing quality for people of all ages but is particularly important for mobility-restricted individuals to enable independence ( Nowossadeck *et al*., 2023). This recognizes that a vital component of ageing and living *well* in place is being able to leave the home to participate and engage in a broader than home environment ( Grove, 2021).

## The consequences of inadequate housing and waiting for regeneration

This paper has revealed four key health concerns that have arisen from living in inadequate social housing, which interlink the physical, emotional, and psychosocial dimensions.

In the first instance, the presence of mould and damp conditions, poor ventilation, and overcrowding have led many homes to experience indoor air quality issues, that have a direct impact on residents’ physical health, in particular their respiratory health. 

Beyond the home, social safety concerns due to drug dealing and anti-social behaviour have resulted in stress, anxiety, depression, fear, and ultimately, avoidance behaviour, with many residents afraid to let their children play in communal spaces and embarrassing to invite people over. This, in turn, has limited their social networks and their diminished their ability to obtain health and well-being through social means. Whilst ‘networks of care’ ( Manzo, 2022b) between some residents are strong, particularly amongst the women, everyday encounters with strangers are fraught.

A lack of green spaces and play areas for children, which are vital components of healthy and restorative placemaking ( Roe & McCay, 2021), means that residents are unable to benefit from being outdoors and engaging with high-quality green spaces, which are known to provide a range of public health benefits ( Gianfredi *et al*., 2021; Mitchell & Popham, 2007). Existing research has shown that green spaces play an important role in stress mitigation and health support, particularly in low-income urban communities ( Ward Thompson *et al*., 2016). Instead, within Oliver Bond, a combination of anti-social behaviour, criminal activities, and limited green spaces has created an unsafe immediate environment for residents. Although certain enhancements have been realized following the establishment of Bridgefoot Street park, as detailed in an earlier section, along with an increase in the local police force 'Garda' presence, including daily patrols and undercover operations targeting drug-related activities, these measures have yet to fully rectify the issue of anti-social behaviour and crime within the Oliver Bond area.

Inhabitants of substandard housing conditions not only grapple with the immediate physical challenges posed by their living spaces, but also face complex emotional and social repercussions. Physical barriers such as multiple flights of stairs have made it exceedingly difficult for certain residents to leave their homes. Consequently, they find themselves unable to actively engage and participate in their broader neighbourhoods and communities. For many, safety concerns and the absence of available and accessible infrastructure force them into a state of social withdrawal, essentially trapping them in housing that is detrimental to their health. This predicament extends beyond physical realms. Metaphorically, these individuals find themselves ‘stuck.’ They are stuck in housing conditions that threaten their well-being; however, they are also stuck in a state of limbo, grappling with a sense of powerlessness.

A significant element of the broader context surrounding these results, is the residents' knowledge that they are facing at least a two-decade wait before the long-promised regeneration programme can be completed. While the survey may not have directly probed this aspect, the media campaign and quotations therein suggest that the prolonged waiting period and lack of control over their circumstances inflict health consequences on these individuals. The prolonged delay in housing improvement generates additional ontological insecurity, as residents feel threatened by the inaction and their lack of control, which extends for an additional 20 years. The concept of ontological insecurity directly affects housing stability, with these factors collectively undermining the feeling of being ‘at home.’ Individuals struggle to establish roots and a sense of belonging within their residences, making it difficult to cultivate a deep connection with their living environment. Stress levels in inadequate housing are exacerbated when the uncertainty introduced by housing maintenance shortcomings and unaddressed repairs ‘spills over’ ( Bolger *et al*., 1989) into increased emotional and relational distress within households. High stress levels can lead to more negative perspectives and erode the sense of ontological safety ( Giddens, 1992), rendering social housing tenants particularly vulnerable to the stress of awaiting the regeneration of their flats. Furthermore, the stress and distress arising from this situation can be attributed to structural factors, particularly the feeling of being in a state of limbo. Stress stems from an inability to control the situation, especially when necessary repairs are the responsibility of others who fail to fulfil their obligations. This further undermines the sense of security and contributes to a sense of not feeling at home in their residences, as they grapple with the uncertainty of when change will occur and the powerlessness they experience ( Byrne & McArdle, 2022).

## Addressing the health impact and policy landscape

To address the health impact of inadequate housing, it is imperative that our housing policy comprehensively recognizes the profound health determinants associated with housing and seamlessly integrates housing and health.

### Exploring the Current Policy Landscape

The Dublin City Council has taken initial steps to acknowledge the pressing need for deep renovation and retrofitting within a comprehensive area-based renewal program, aimed at addressing dereliction in these housing areas. The city has been actively engaged in providing responsive repairs, maintenance services, heating system upgrades, and replacements for fixtures and fittings. Additionally, the city is aligning with major plans under the EU renovation wave, focusing on climate action adaptations that decrease carbon-based energy consumption, while enhancing thermal efficiency and overall comfort. However, the backlog of disrepair in older social housing remains substantial and its adverse effects on daily living persist. When social housing complexes intersect with local urban dereliction, the overall deterioration of the quality of the living environment can pose significant threats to health and well-being.

### Challenges and the Path Forward

The challenge lies in envisioning a society that is deeply sensitive to health and well-being, crafting a housing system capable of sustaining it, and strategically implementing necessary measures to realize this vision ( Smith, 2012). Regeneration and broader area improvements in Ireland constitute a complex process, involving elements that fall beyond the jurisdiction of the Dublin City Council. These include key decisions regarding capital expenditure and policymaking that remain under the purview of the national government. Nevertheless, Dublin City's Local Community Development Committee (LCDC) actively supports a Community of Practice (CoP), comprising practitioners experienced in delivering housing services, including care and support services, particularly those with prior experience in area-based estate regeneration programs. Moreover, the city fosters regeneration forums involving local stakeholders, organized to aid the city council throughout the regeneration process currently in development. For the residents of the Oliver Bond House, securing the right to adequate housing is paramount.

### The imperative of embedding an ethics of care in policy

There are hopeful indications that the Dublin City Council forges connections among housing, health, well-being, and community development within the policy landscape. A workshop ^6^ was conducted in Dublin in 2022, underscoring the collaborative approach of the City of Care. However, the vital question remains: Without the embedding of an ethics of care, as exemplified by the 'City of Care' framework, or the incorporation of health into housing policy, it's unlikely that the health and well-being of residents will take precedence through the urgent restoration and enhancement of social housing quality.

## How can ethics of care and resident health be prioritized into housing policy?

The fundamental question raised in this study is how to prioritize and integrate the principles of ethics of care, particularly responsibility and well-being of residents, into housing policy.

### Policy recommendations

Our findings have highlighted the urgent need to place greater priority on the maintenance of existing social housing stock. They demonstrate the imperative for public housing policies to recognise both the quality and quantity of adequate housing provision, where care is at the heart of housing policies. To address these imperatives, we introduce the innovative 'City of Care' framework, designed to embed an ethics of care within cities. This framework champions principles of public health, community well-being, solidarity, resident empowerment, and social justice at the forefront. Given the critical role housing plays in public health, now is the time for a joint public housing and public health agenda. Such an approach is essential for creating healthier homes and tackling broader social inequalities stemming from the daily repercussions of inadequate housing.

The 'City of Care' framework comprises four essential components:

**1.   Acknowledging the existing Carelessness Crisis within the Housing context**: A call for Dublin to become a caring city, recognizing the urgency of this transformation.

**2.   Promoting Health in All Policies (HiAP)**: Advocating for the integration of health and housing policies to ensure holistic well-being.

**3.   Empowering Community Infrastructure**: Fostering collective agency and women-led social support networks to advocate for improved living conditions.

**4.   Prioritising Affordable Housing**: Advocating for a housing policy that prioritises both the quality and quantity of adequate housing, with a strong focus on public health and social justice.

To effectively integrate health and housing into policy, we propose adopting a Health in All Policies' (HiAP) approach. HiAP recognizes that health disparities are influenced by factors beyond the conventional health sector and its policies ( Braubach, 2011; Sharpe *et al*., 2018). The embedding of health within all policies is vital, as it enables us to address policies influencing transport, housing, urban planning, the environment, education, agriculture, finance, taxation, and economic development, with the aim of promoting overall health and health equity ^7^.

**Figure 12.**
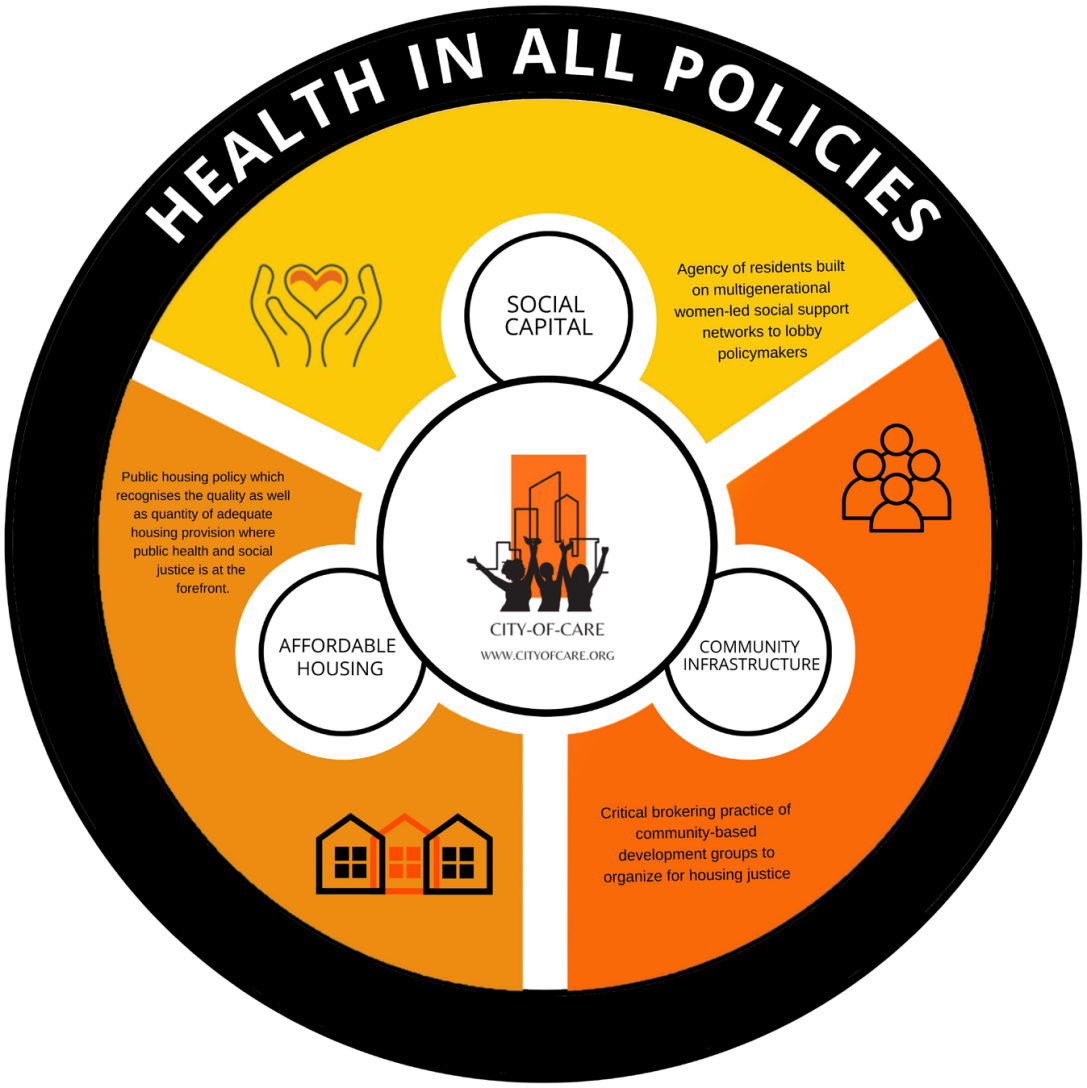
CITY-OF-CARE ‘Health in All Policies’ conceptual diagram. Source: Manzo and Grove (2023).

In conclusion, there is a pressing need to prioritize care, health, and well-being as fundamental principles in all policies. The 'City of Care' framework, in conjunction with a participatory approach, empowers residents and fosters cooperation among inter-sectoral stakeholders, paving the way for integrated health and housing policies that prioritize the well-being of all. Figure 12 reimagines the 'City of Care' framework within the context of 'Health in All Policies,' encompassing three interconnected spheres. As evident throughout our work, social capital embodies residents' collective agency, empowered by multigenerational women-led social support networks that advocate for improved living conditions. Affordable housing represents a housing policy that places equal emphasis on the quality and quantity of adequate housing with a special focus on public health and social justice. The community infrastructure highlights the pivotal role of community-based development groups in facilitating housing justice initiatives.

## Ethics and consent

This study was granted Ethical approval by the EU Commission Ethical Review Board on 9 ^th^ April 2021, as well as approval from the Research Ethics Committee of the University of Milan, Italy, on 14 ^th^ December 2020 (approval number: 119/20). Informed written informed consent was obtained from all participants, using consent forms.

## Data Availability

The full data generated and analysed during the current study cannot be sufficiently de-identified and therefore cannot be made publicly available. This restriction is due to ethical considerations, given the highly vulnerable nature of the social housing communities under study, including aspects related to crime and individuals involved in criminal activities. Disclosing this information could pose a risk to both residents and researchers.

## Footnotes

^1^ This project has received funding from the European Commission Horizon 2020 Marie Skłodowska Curie Individual Fellowship (G.A.890603). See www.cityofcare.org and https://cordis.europa.eu/project/id/890603

^2^ “Residents living with mould and damp campaign for fast-track regeneration”, article by Jade Wilson published on *The Irish Times*, June 30 ^th^ 2021.

^3^ Dublin Corporation, known by generations of Dubliners simply as The Corpo, is the former name given to the city government and its administrative organisation in Dublin between the twelfth century and 1 January 2002. It is now known as Dublin City Council.

^4^ The Oliver Bond Regeneration Forum is a representative structure for proposed physical and social regeneration of Oliver Bond House. Its membership comprises resident representatives, local Councillors and senior personnel from Dublin City Council and other relevant statutory agencies.

^5^ One person completed this survey per household.

^6^ ‘Care, Health and Housing’ collaborative workshop, organized by Lidia K.C. Manzo on October 18, 2022 at the Digital Hub in Dublin 8, Ireland.

^7^ Available at: https://www.who.int/activities/promoting-health-in-all-policies-and-intersectoral-action-capacities (Accessed 30th October 2023).
