# Supplementary material for: Inadequate Social Housing and Health: The Case of Oliver Bond House, The Liberties, Dublin
Source: Open Res Eur. 2024 Mar 13;3:211. Originally published 2023 Nov 28. [Version 2] doi: 10.12688/openreseurope.16767.2 (PMC10879759; doi:10.12688/openreseurope.16767.2)
Supplement: Peer review history

# Reviewer report: Reviewer response for version 2

**DOI:** 10.21956/openreseurope.18791.r38907

**Published:** 2024-05-06

**Authors:** Declan Redmond

**Affiliations:** 1 School of Architecture, Planning and Environmental Policy, University College Dublin, Dublin, Leinster, Ireland

No comments

If applicable, is the statistical analysis and its interpretation appropriate?

Not applicable

Is the study design appropriate and is the work technically sound?

Yes

Is the work clearly and accurately presented and does it engage with the current literature?

Yes

Are the conclusions drawn adequately supported by the results?

Yes

Are sufficient details of methods and analysis provided to allow replication by others?

Yes

Are all the source data and materials underlying the results available?

Yes

Reviewer Expertise:

social housing policy; urban regeneration; planning policy

I confirm that I have read this submission and believe that I have an appropriate level of expertise to confirm that it is of an acceptable scientific standard.

# Reviewer report: Reviewer response for version 2

**DOI:** 10.21956/openreseurope.18791.r38909

**Published:** 2024-04-22

**Authors:** Nicholas Pleace

**Affiliations:** 1 Centre for Housing Policy, University of York, York, England, UK

No further comments

If applicable, is the statistical analysis and its interpretation appropriate?

Not applicable

Is the study design appropriate and is the work technically sound?

Yes

Is the work clearly and accurately presented and does it engage with the current literature?

Yes

Are the conclusions drawn adequately supported by the results?

Yes

Are sufficient details of methods and analysis provided to allow replication by others?

Yes

Are all the source data and materials underlying the results available?

Yes

Reviewer Expertise:

Housing exclusion, homelessness, inequalities in housing and area effects.

I confirm that I have read this submission and believe that I have an appropriate level of expertise to confirm that it is of an acceptable scientific standard.

# Reviewer report: Reviewer response for version 1

**DOI:** 10.21956/openreseurope.18113.r36858

**Published:** 2024-02-22

**Authors:** Erin Ruel

**Affiliations:** 1 College of Arts & Sciences, Georgia State University, Atlanta, Georgia, USA

This paper is well written with a strong argument and builds on existing research. The results themselves are quite basic in terms of analysis. Rather than going deeper in terms of analysis, the authors tend to repeat themselves. The findings are supported, however. I like the health in all policies suggestion. While much of the impact of housing on health is indirect, it is important to recognize how policy in one area impinges on other areas.

If applicable, is the statistical analysis and its interpretation appropriate?

Yes

Is the study design appropriate and is the work technically sound?

Yes

Is the work clearly and accurately presented and does it engage with the current literature?

Yes

Are the conclusions drawn adequately supported by the results?

Yes

Are sufficient details of methods and analysis provided to allow replication by others?

Partly

Are all the source data and materials underlying the results available?

Partly

Reviewer Expertise:

health, housing, survey methods

I confirm that I have read this submission and believe that I have an appropriate level of expertise to confirm that it is of an acceptable scientific standard.

# Response

**Published:** 2024-03-08

**Authors:** Lidia Katia C. Manzo

**Affiliations:** Languages, Literatures, Cultures and Mediations, University of Milan, Milan, Lombardy, Italy

Thank you very much for your thoughtful comments and for dedicating time to review our paper. We appreciate your insights and agree with your observation regarding the level of analysis in the findings. Given the urgency of the health situation for residents, our primary aim with this paper was to present preliminary results from the survey. However, we acknowledge the importance of delving deeper into the analysis, and we will certainly consider this for future iterations of our research. Regarding the participatory nature of our research, we felt compelled to share our preliminary findings with the wider community. We believe that transparency and engagement with stakeholders are crucial aspects of our work, and we hope that by sharing our initial results, we can stimulate further discussion and collaboration in this important area. We are delighted to hear that you found the 'health in all policies' suggestion appealing. Our hope is that this concept will prove valuable for policymakers, encouraging them to integrate considerations of care, health, and wellbeing across all sectors of their work. Once again, we appreciate your feedback and constructive criticism, which will undoubtedly strengthen the quality and impact of our research. Thank you for your time and consideration.

# Reviewer report: Reviewer response for version 1

**DOI:** 10.21956/openreseurope.18113.r37867

**Published:** 2024-02-20

**Authors:** Declan Redmond

**Affiliations:** 1 School of Architecture, Planning and Environmental Policy, University College Dublin, Dublin, Leinster, Ireland

This is a comprehensive and detailed account of how poor housing conditions are inextricably linked to all manner of negative health outcomes. The case study is of a social housing flat complex, named Oliver Bond, located in Dublin's inner city. The paper situates the case study in conceptual terms by examining literature about housing and health but also by introducing the idea of an ethics of care. This idea of an ethic of care, or a city of care framework, is novel in Irish terms, but it fits the case study very well. The paper uses the existing literature to very good effect.

 The methods employed, dominated by an ethnographic approach, are appropriate and indeed work very well in generating very interesting results. In fact the results, which are well described, using relevant quotations from residents, are quite depressing. Negative physical and mental health impacts are clearly and movingly described. This is made more impactful when reference is made to the 'managed decline' of the neighbourhood and a possible 20 year wait for regeneration. It is no wonder that many residents feel 'stuck' in place and are sceptical of improvement given the long time envisaged for area regeneration.

 This is a really interesting paper and a corrective to some of the self-congratulation that is sometimes seen in Ireland with regard to economic prosperity. Given Ireland is a relatively prosperous country why have places like Oliver Bond been left behind? The 'city of care framework' makes sense but will the response of the state be adequate?

If applicable, is the statistical analysis and its interpretation appropriate?

Not applicable

Is the study design appropriate and is the work technically sound?

Yes

Is the work clearly and accurately presented and does it engage with the current literature?

Yes

Are the conclusions drawn adequately supported by the results?

Yes

Are sufficient details of methods and analysis provided to allow replication by others?

Yes

Are all the source data and materials underlying the results available?

Yes

Reviewer Expertise:

social housing policy; urban regeneration; planning policy

I confirm that I have read this submission and believe that I have an appropriate level of expertise to confirm that it is of an acceptable scientific standard.

# Response

**Published:** 2024-02-20

**Authors:** Lidia Katia C. Manzo

**Affiliations:** Languages, Literatures, Cultures and Mediations, University of Milan, Milan, Lombardy, Italy

Thank you sincerely for dedicating your time and effort to conduct a thorough review and provide insightful feedback, which highlighted crucial aspects of the paper.

# Reviewer report: Reviewer response for version 1

**DOI:** 10.21956/openreseurope.18113.r36482

**Published:** 2024-01-16

**Authors:** Nicholas Pleace

**Affiliations:** 1 Centre for Housing Policy, University of York, York, England, UK

This is an interesting and thorough study which has established a clear conceptual and methodological framework to explore the intersections of place, housing and health. The use of resident participation through a survey has generated some interesting results, I'm not quite sure that the study is really an ethnography, nor is it straightforwardly qualitative (in the sense that there are no interviews or focus groups reported, instead the emphasis is on what appears to be a text-based survey).  Nevertheless, the results from using this methodology are clearly and well presented and while in some senses the research tells us nothing really new (these sorts of links between housing, community and environmental conditions and public health are well documented and were part of the original rationale for developing social/public housing in Ireland and elsewhere in Europe) all the points made here remain valid, important and urgent. The broad associations between housing, area effects and physical and mental health, while widely acknowledged, receive less attention than research that explores links between behaviour and health. It is refreshing to see robust research that looks at the impacts of housing, community and place on physical and mental health and which does not attempt to explain differences in health and wellbeing in simply terms of individual behaviour and 'cultures' that supposedly encourage unhealthy eating, smoking, drug use etc. The research is useful in exploring how people recognise that the places in which they live are unhealthy and that in order to address the concerns around housing, area effects and health, public policy intervention is necessary.

If applicable, is the statistical analysis and its interpretation appropriate?

Not applicable

Is the study design appropriate and is the work technically sound?

Yes

Is the work clearly and accurately presented and does it engage with the current literature?

Yes

Are the conclusions drawn adequately supported by the results?

Yes

Are sufficient details of methods and analysis provided to allow replication by others?

Yes

Are all the source data and materials underlying the results available?

Yes

Reviewer Expertise:

Housing exclusion, homelessness, inequalities in housing and area effects.

I confirm that I have read this submission and believe that I have an appropriate level of expertise to confirm that it is of an acceptable scientific standard.

## References

[1] : Inadequate Social Housing and Health: The case of Oliver Bond House in inner-city Dublin. *Open Research Europe* .2023;3: 10.12688/openreseurope.16767.1 10.12688/openreseurope.16767.1 PMC1087975938384817

# Response

**Published:** 2024-01-17

**Authors:** Lidia Katia C. Manzo

**Affiliations:** Languages, Literatures, Cultures and Mediations, University of Milan, Milan, Lombardy, Italy

Thank you sincerely for the insightful review and valuable feedback that has significantly contributed to clarifying key aspects of the paper. We are truly grateful for the time and effort you invested in thoroughly evaluating the content. It's important to highlight that this article draws from a broader project designed to incorporate participatory research and ethnographic practices at all stages, especially when dealing with vulnerable populations. The trust established through negotiations with the community of our case study was indispensable for gathering the survey data. Currently, we are actively working on other papers focusing on the qualitative aspects of this research. As you astutely noted, addressing concerns related to housing, area effects, and health necessitates public policy intervention. Consequently, we feel compelled to share these survey results on this EU open access journal to foster a meaningful public discourse.
