# Supplementary material for: Inadequate Social Housing and Health: The case of Oliver Bond House in inner-city Dublin
Source: Open Res Eur. 2023 Nov 28;3:211. [Version 1] doi: 10.12688/openreseurope.16767.1 (PMC10879759; doi:10.12688/openreseurope.16767.1)
Supplement: Peer review history

# Reviewer report: Reviewer response for version 1

**DOI:** 10.21956/openreseurope.18113.r37872

**Published:** 2024-06-05

**Authors:** Roberta Cucca

**Affiliations:** 1 Department of Urban and Regional Planning, Norwegian University of Life Sciences, Ås, Norway

The article presents an interesting analysis of health determinants in urban settings, offering insights into an important area of urban studies and housing research. Inadequate housing is indeed a relevant social justice issue that adversely affects health. The article employs and defines the concept of "adequacy," underlining several factors that must be considered in determining whether particular forms of housing can be considered "adequate” or not.  Methodologically, the study is robust and well-structured. It combines personal network analysis and visualization with ethnographic and participatory techniques, allowing for the creative integration of qualitative research and network analysis by employing a resident-led survey to collect data from key informants. The findings, while not surprising, underscore the need for policy action to address health disparities in urban communities. Overall, the article contributes to the literature on urban health and housing, especially in its potential to inform future research and policy agendas. It calls for public sociology that bridges professional and policy sociology, with critical sociology.

If applicable, is the statistical analysis and its interpretation appropriate?

Not applicable

Is the study design appropriate and is the work technically sound?

Yes

Is the work clearly and accurately presented and does it engage with the current literature?

Yes

Are the conclusions drawn adequately supported by the results?

Yes

Are sufficient details of methods and analysis provided to allow replication by others?

Yes

Are all the source data and materials underlying the results available?

Yes

Reviewer Expertise:

urban studies

I confirm that I have read this submission and believe that I have an appropriate level of expertise to confirm that it is of an acceptable scientific standard.
